# Subunit composition of the human cytoplasmic dynein-2 complex

**DOI:** 10.1242/jcs.159038

**Published:** 2014-11-01

**Authors:** David Asante, Nicola L. Stevenson, David J. Stephens

**Affiliations:** Cell Biology Laboratories, School of Biochemistry, Medical Sciences Building, University of Bristol, Bristol BS8 1TD, UK

**Keywords:** Microtubule motor, Dynein, Cilia, Intraflagellar transport

## Abstract

Cytoplasmic dynein-2 is the motor for retrograde intraflagellar transport (IFT), and mutations in dynein-2 are known to cause skeletal ciliopathies. Here, we define for the first time the composition of the human cytoplasmic dynein-2 complex. We show that the proteins encoded by the ciliopathy genes *WDR34* and *WDR60* are bona fide dynein-2 intermediate chains and are both required for dynein-2 function. In addition, we identify TCTEX1D2 as a unique dynein-2 light chain that is itself required for cilia function. We define several subunits common to both dynein-1 and dynein-2, including TCTEX-1 (also known as DYNLT1) and TCTEX-3 (also known as DYNLT3), roadblock-1 (also known as DYNLRB1) and roadblock-2 (also known as DYNLRB2), and LC8-1 and LC8-2 light chains (DYNLL1 and DYNLL2, respectively). We also find that NudCD3 associates with dynein-2 as it does with dynein-1. By contrast, the common dynein-1 regulators dynactin, LIS1 (also known as PAFAH1B1) and BICD2 are not found in association with dynein-2. These data explain why mutations in either WDR34 or WDR60 cause disease, as well as identifying *TCTEX1D2* as a candidate ciliopathy gene.

## INTRODUCTION

Normal cell function depends on the accurate movement and positioning of intracellular organelles and protein complexes. Microtubule motor proteins are crucial to these processes, with the cytoplasmic dyneins being the primary minus-end-directed microtubule motors. Two cytoplasmic dynein complexes exist in mammalian cells; dynein-1 is the better characterized of these, being involved in membrane traffic, organelle dynamics and chromosome segregation during mitosis (Paschal and Vallee, 1987). By contrast, the cytoplasmic dynein-2 complex mediates the retrograde component of intraflagellar transport (IFT), the process by which primary and motile cilia as well as flagella function (Gibbons et al., 1994; Criswell et al., 1996). Retrograde IFT is essential for normal developmental signaling, notably the Hedgehog pathway in which dynein-2 acts in concert with the IFT-A complex to drive the transport of activated components of this pathway from the cilia tip to the cell body (Huangfu and Anderson, 2005; Liu et al., 2005; May et al., 2005). Furthermore, IFT-A is known to be required for the loading of key components into cilia (Mukhopadhyay et al., 2010; Liem et al., 2012). Defects in the function of primary cilia lead to a cohort of human diseases known as the ciliopathies (Waters and Beales, 2011); among these, mutations in components of the dynein-2 complex cause Jeune syndrome, short rib polydactyly type III and asphyxiating thoracic dystrophy (Dagoneau et al., 2009; Huber et al., 2013; McInerney-Leo et al., 2013; Schmidts et al., 2013b; Schmidts et al., 2013a).

Dynein-1 is comprised of a dimer of heavy chain subunits (encoded by the *DYNC1H1* gene), which associate with two copies of an intermediate chain (DYNC1I1 or DYNC1I2, depending on the tissue type), two copies of one of two light intermediate chains (DYNC1LI1 or DYNC1LI2) and a number of light chain subunits [including the dynein light chain (DYNLL1), roadblock (DYNLRB1 and DYNLRB2) and Tctex families (DYNLT1, DYNLT3) (Pfister et al., 2005; Wickstead and Gull, 2007; Kardon and Vale, 2009)]. The light chains are required for the correct assembly of the dynein complex and have been implicated in controlling its association with cargo molecules. For the majority of its functions, dynein-1 also associates with one or more binding partners (Kardon and Vale, 2009; Splinter et al., 2012). The best described of these are the dynactin complex, lissencephaly-1 protein (LIS1, also known as PAFAH1B1) and bicaudal gene products (notably BICD2) (Splinter et al., 2012). By contrast, the molecular composition of the dynein-2 complex, particularly in mammals, is not well defined.

Although specific genes encoding a dynein-2-specific heavy chain [DYNC2H1, also known as DHC1B and DHC2 (Criswell et al., 1996)] and light intermediate chain [DYNC2LI1, also known as LIC3 (Grissom et al., 2002)] have been identified, the full subunit composition of the motor and biochemical characterization of the subunit composition in metazoans is lacking. Model organisms such as *Chlamydomonas* and *Tetrahymena* have provided further clues that equivalents to the other known dynein-1 subunits are also present. *Chlamydomonas* genes encoding FAP133 and FAP163 (orthologs of mammalian WDR34 and WDR60, respectively) encode functional intermediate chains of algal dynein-2 (Rompolas et al., 2007; Patel-King et al., 2013). FAP133 was shown to have putative LC8 (also known as DYNLL1 and DYNLL2 in humans) binding motifs and to localize around the basal body and within the flagellum (Rompolas et al., 2007). Furthermore, LC8 is known to play roles outside of the context of the dynein-1 complex, which could explain some of the FAP133/WDR34 data to date. Our own work has shown that WDR34 localizes to the pericentrosomal region and is required for ciliogenesis and proper cilia function *in vitro* (Asante et al., 2013). We have also previously defined a role for the dynein light chain Tctex-1 in controlling cilia length, presumably in association with dynein-2 (Palmer et al., 2011). Others have also shown that WDR34 localizes to a pericentrosomal region and that a fluorescent protein fusion of WDR34 (WDR34–tGFP) is present in cilia (Schmidts et al., 2013a). Furthermore WDR34–tGFP co-immunoprecipitates with FLAG-tagged LC8, consistent with a role for WDR34 in dynein-2 function.

FAP163 has also been shown to localize to the flagellar matrix, to co-purify with FAP133 and LC8, and, in the planarian *Schmidtea mediterranea*, to be absolutely required for ciliogenesis (Patel-King et al., 2013). Recent work from several groups has shown that mutations in the human orthologs of FAP133 (WDR34) and FAP163 (WDR60) cause skeletal ciliopathies (Huber et al., 2013; McInerney-Leo et al., 2013; Schmidts et al., 2013a). Although these data are consistent with WDR34 and possibly WDR60 being key components of human dynein-2, biochemical data validating this are lacking. It also remains unclear whether dynein-2 functions in cooperation with dynactin or other dynein-1 accessory complexes. LIS1, for example, is known to function in concert with both cytoplasmic dynein-1 (Faulkner et al., 2000) and the axonemal dyneins (Pedersen et al., 2007). Because a metazoan dynein-2 complex has not been described in detail, we set out to define the subunit composition of the human dynein-2 complex and to determine whether known dynein-1 regulators also modulate the function of this poorly characterized second cytoplasmic dynein motor.

## RESULTS

### mGFP–WDR34 localizes to cilia

**Fig. 1.**
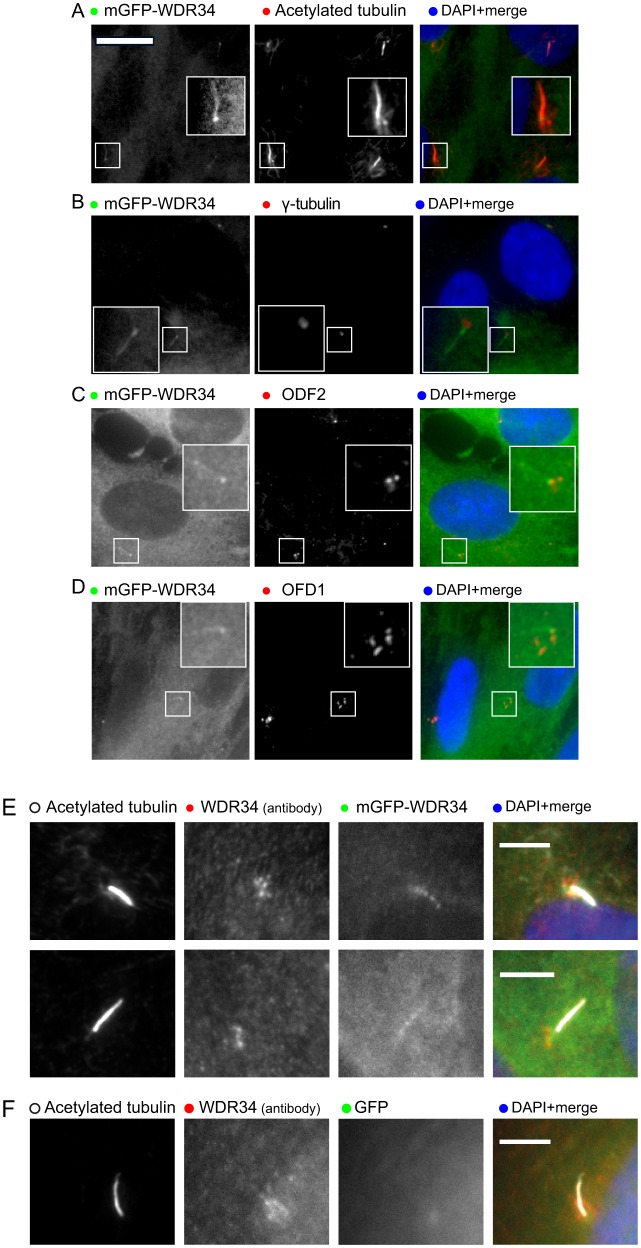
**Localization of mGFP–WDR34.** (A) mGFP–WDR34 (green) localizes to a cytosolic pool and to primary cilia, colocalizing with acetylated tubulin (red). Labeling of (B) γ-tubulin, (C) ODF2 and (D) OFD1 (all red) demonstrate the accumulation of mGFP–WDR34 at the base of the cilium with some labeling evident along its length. Insets show enlargements of the areas indicated in the main images. (E) Cells expressing mGFP–WDR34 were fixed with paraformaldehyde and labeled to detect acetylated tubulin and WDR34, as indicated. Two examples are shown. (F) Cells expressing GFP were processed as in E to demonstrate an absence of GFP from primary cilia. Scale bars: 5 µm.

We generated a human telomerase-immortalized retinal pigment epithelial (hTERT-RPE1) cell line stably expressing WDR34 fused to a monomeric form of GFP (mGFP). Fig. 1 shows that mGFP–WDR34 localizes to centrosomes and primary cilia in serum-starved cells as well as showing a diffuse cytoplasmic distribution. This localization to cilia was confirmed by co-labeling with antibodies directed against acetylated tubulin (Fig. 1A). mGFP–WDR34 also colocalizes at the base of the cilium with known components of the basal body (γ-tubulin, ODF2 and OFD1, Fig. 1B–D). Fig. 1E shows that the localization of mGFP–WDR34 within cilia differs from that of the endogenous protein, which localizes to pericentrosomal structures; Fig. 1F shows that the localization of mGFP–WDR34 to cilia is specific, as GFP alone is not localized in cilia.

### Proteomics reveals mGFP–WDR34 to be a component of the dynein-2 complex

We sought to use this cell line to define the other proteins associated with WDR34. To achieve this, we took a proteomic approach comparing the interactome of mGFP–WDR34 with that of mGFP using a GFP-nanotrap (Rothbauer et al., 2006). Supplementary material Table S1 shows the proteomics data for all known dynein-1 and putative dynein-2 components that were identified in this experiment. Supplementary material Table S1A shows the highly efficient isolation of mGFP–WDR34 using this approach. Large numbers of peptides and spectra were also seen for WDR60 as well as the known dynein-2 heavy chain (DYNC2H1). We also detected (albeit at low abundance) the known dynein-2 light intermediate chain subunit DYNC2LI1/LIC3. Listed in supplementary material Table S1B are the dynein light chains that we identified. Here, all known dynein-1 light chain isoforms (LC8-1 and LC8-2, Tctex-1 and Tctex-3, and roadblock-1 and roadblock-2) were identified in association with mGFP–WDR34. In addition, we detected TCTEX1D2 (also known as Tctex-2). This subunit is not found in association with mGFP alone. We also noted (supplementary material Table S1C) the association of mGFP–WDR34 with two regulators of dynein-1 function, NudC and NudCD3 (also called NudC-like or NUDCL). Supplementary material Table S1D shows the data for the key dynein accessory factors that were detected in our proteomics analysis. These show that mGFP–WDR34 does not associate with dynactin, bicaudal 1 or LIS1 at levels above that of mGFP alone. Bicaudal 2, Spindly, NudE (also known as NDE1) and Nudel (also known as NDEL1), all of which are known accessory factors for dynein-1, were not detected at all. We then used these proteomic data to guide our further biochemical analyses.

### Validation of the components and interactions of the human dynein-2 complex

**Fig. 2.**
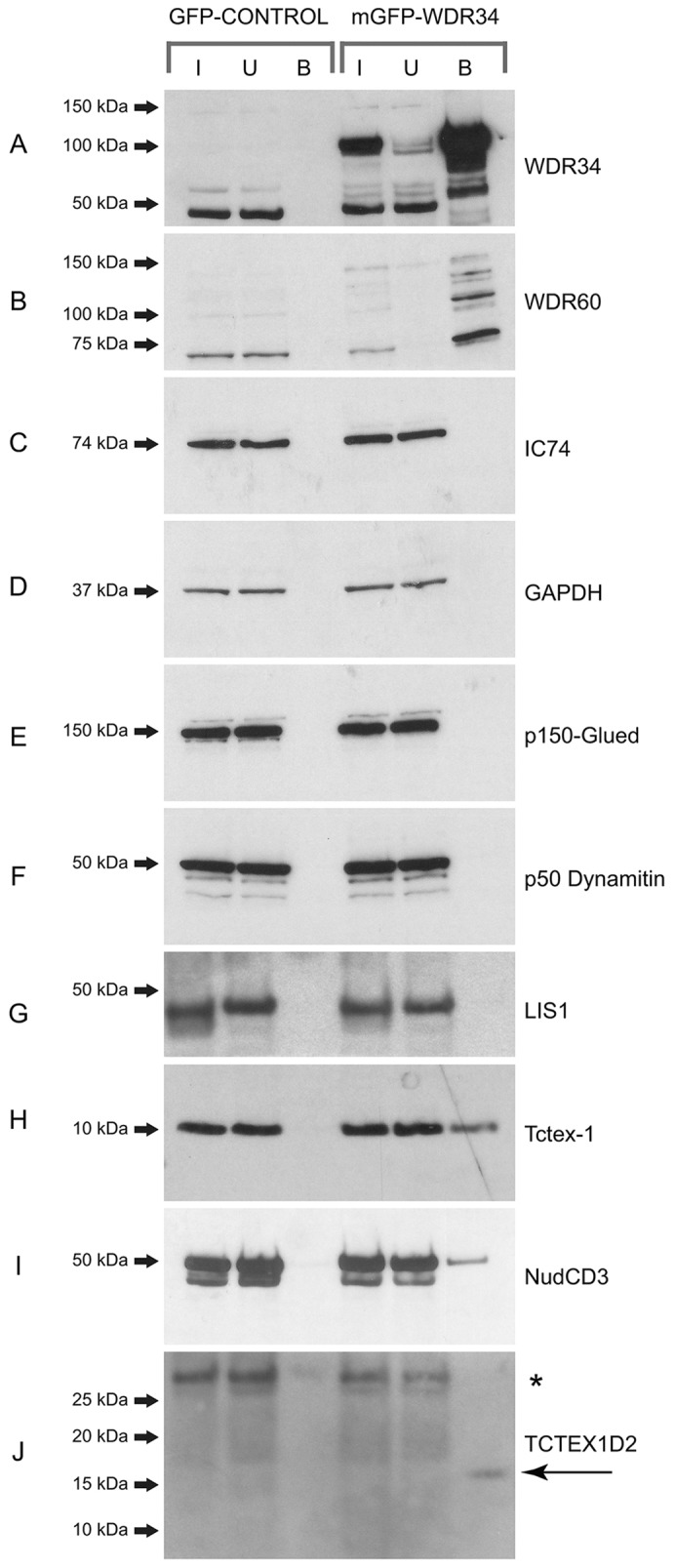
**Immunoprecipitation from serum-starved RPE1 cells stably expressing either mGFP or mGFP–WDR34.** GFP-traps of cells expressing mGFP alone or mGFP–WDR34 were separated by SDS-PAGE and immunoblotted to detect (A) WDR34, (B) WDR60, (C) IC74 (DYNC1I2), (D) GAPDH, (E) p150^Glued^, (F) p50^dynamitin^, (G) LIS1, (H) Tctex-1, (I) NudCD3 or (J) TCTEX1D2, as indicated. Molecular-mass markers are shown (kDa). In each case, the lanes show the input (‘I’), the unbound fraction (‘U’) and the bound fraction (‘B’), as indicated. The asterisk in J indicates non-specific bands.

The above data indicate that both WDR34 and WDR60 are intermediate chains of the dynein-2 complex. These findings were then validated using immunoblotting. Fig. 2A shows the specific isolation of mGFP–WDR34. Importantly, we found that the endogenous WDR34 did not co-immunoprecipitate with mGFP–WDR34, suggesting that the recombinant form does not form a complex with the endogenous protein. Immunoblotting for WDR60 showed that this was highly efficiently isolated in complex with mGFP–WDR34 (Fig. 2B), implying that these two putative intermediate chains might be present in the same complex. As expected from the proteomic data, the canonical dynein-1 intermediate chain, IC74 (also known as DYNC1I2), was not immunoprecipitated with mGFP–WDR34 (Fig. 2C), nor was a negative control protein, GAPDH (Fig. 2D). Two components of the dynactin complex, p150^Glued^ (Fig. 2E) and p50^dynamitin^ (Fig. 2F) (also known as DCTN1 and DCTN2, respectively) also failed to immunoprecipitate with mGFP–WDR34; similarly, LIS1 was not detected (Fig. 2G). By contrast, Tctex-1 (Fig. 2H) and NudCD3 (Fig. 2I) were both efficiently isolated. Consistent with our identification of TCTEX1D2 in our proteomics, we were able to identify TCTEX1D2 in association with mGFP–WDR34 (Fig. 2J). TCTEX1D2 is not detectable in whole-cell lysates but the enrichment following GFP-nanotrap isolation of mGFP–WDR34 enables its detection in immunoprecipitates (Fig. 2J). These data reveal some specificity, because both Tctex-1 and NudCD3, but not TCTEX1D2, associate with dynein-1, as determined in five independent pull downs of dynein-1; dynactin, LIS1 and BICD2 were also always identified in these dynein-1 pull downs from both HeLa and RPE-1 cells (data not shown). We have been unable to analyze the dynein-2 heavy chain or light intermediate chain subunits by immunoblotting, owing to a lack of available antibodies.

**Fig. 3.**
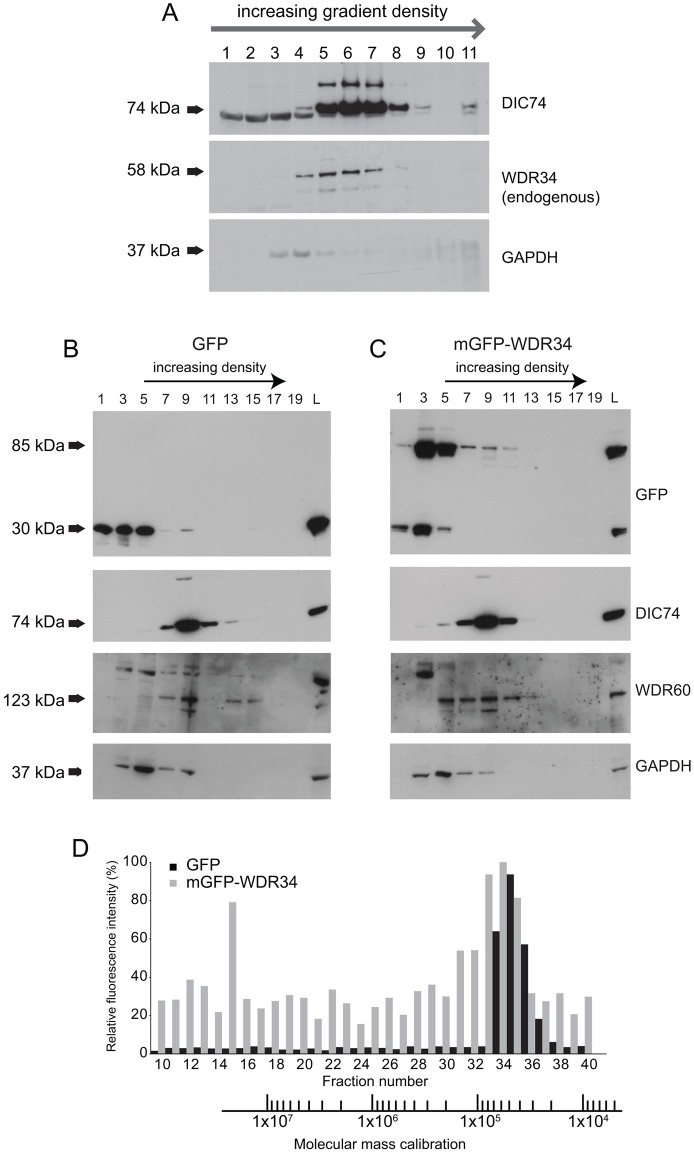
**Sucrose density gradient centrifugation of cell lysates from RPE1 cells.** (A) RPE1 cells were lysed and loaded onto a 5–40% sucrose density gradient. After centrifugation, 1-ml fractions were removed from the gradient and probed by immunoblotting to determine the distribution across the gradient of DIC74 (DYNC1I2), WDR34 or GAPDH. (B) RPE1 cells stably expressing (B) GFP or (C) mGFP–WDR34 were lysed and loaded onto a 5–20% sucrose density gradient. A total of 20 500-µl fractions were collected post-centrifugation and alternate fractions were analyzed by SDS-PAGE and immunoblotting for GFP, DIC74, GAPDH and WDR60. L, whole cell lysate. (D) Gel filtration of lysates from GFP-expressing cells (gray bars) and mGFP–WDR34-expressing cells (black bars). Molecular mass calibration is shown below the graph, aligned to the fraction numbers.

Dynein-2 is predicted to be a large-molecular-mass complex with a high sedimentation coefficient, similar to cytoplasmic dynein-1. As predicted for a large macromolecular complex, we found endogenous WDR34 in a large complex that sedimented on sucrose density gradients in similar fractions to the dynein-1 intermediate chain (Fig. 3A). This suggests that WDR34 functions in the context of a high-molecular-mass complex. As expected, mGFP–WDR34 and WDR60 were both found in large complexes (Fig. 3B,C), consistent with their role as intermediate chains within the same dynein-2 complex. By contrast, mGFP (Fig. 3B), like the majority of the control protein GAPDH, was found predominantly in low-density fractions as expected. GAPDH is known to associate with many other proteins including dynein-1 (Zala et al., 2013), which likely explains the low amounts detected across the higher-density gradient fractions. The expression of mGFP–WDR34 had no significant effect on the distribution of WDR60 across the complex (Fig. 3C). Some unincorporated mGFP–WDR34 was observed, as well as some mGFP itself (Fig. 3C), most likely arising as a result of proteolytic processing of the recombinant protein at the linker site between mGFP and WDR34. This could also explain much of the diffuse cytoplasmic labeling seen in Fig. 1. Further to this, we undertook gel filtration of cell lysates to determine the apparent molecular mass of the mGFP–WDR34-containing complexes. Fig. 3D shows that, using a calibrated Superose 6 column, we found that all detectable mGFP–WDR34 fluorescence eluted with the void volume of the column (Fig. 3D, fraction 15, 7.9 ml) corresponding to high-molecular-mass components (∼1×10^7^ Da). GFP, by contrast, eluted within the range of 4–7×10^4^ Da (Fig. 3D, fractions 33–37). As with the sucrose gradients, it is evident that there is a significant fraction of mGFP–WDR34 (and also mGFP as a degradation product from this) that elutes in low-molecular-mass and low-density fractions. This complicates the analysis of the exact proportion of mGFP–WDR34 that is contained within the dynein-2 complex.

### WDR60 is required for ciliogenesis and cilia function

**Fig. 4.**
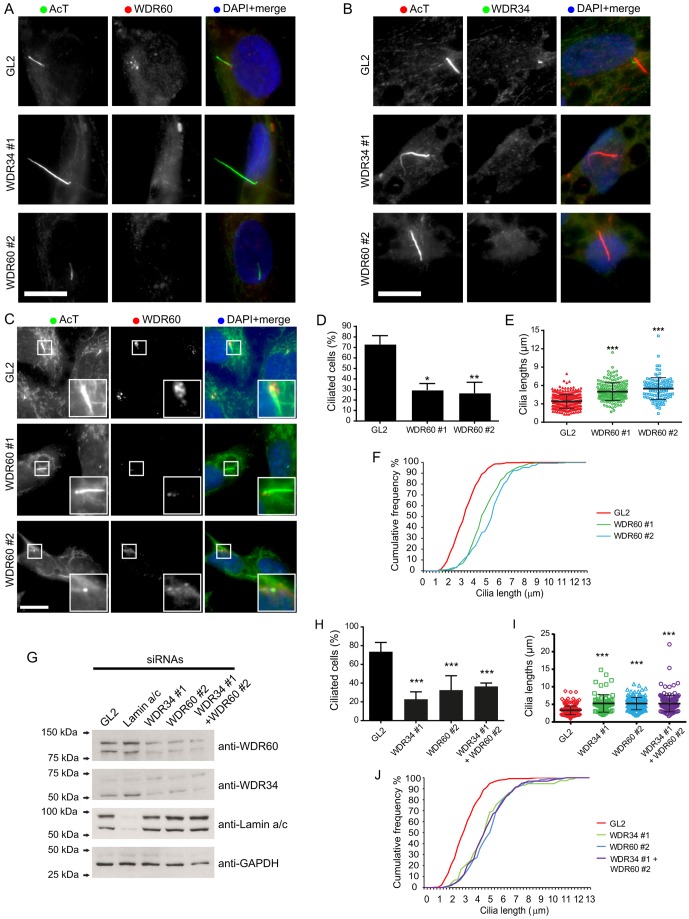
**The stabilities of WDR34 and WDR60 are interdependent.** (A,B) Cells were transfected with siRNA duplexes targeting WDR34 or WDR60, as indicated, and following serum starvation, cells were fixed and immunolabeled to detect acetylated tubulin (AcT) and (A) WDR60 or (B) WDR34. (C) Cells were effectively depleted of WDR60 (shown by loss of immunoreactivity around the centrosome), and labeling showed either longer cilia (example shown for WDR60 #1) or a failure to generate cilia (example shown for WDR60 #2). Insets show enlargements of the areas indicated in the main images. (D) Quantification of the percentage of cells that produced cilia in response to 24-h serum starvation. (E) The lengths of the remaining cilia were measured for control (GL2) or WDR60-depleted cell cultures. (F) Distribution of cilium length represented as a cumulative frequency chart of the percentage of total cilia found in 0.25-µm bins. The data plotted are the same as that shown in E. (G) Immunoblotting confirmed the efficacy of WDR34 and WDR60 siRNAs. Immunoblotting for WDR34 following suppression of WDR60 and vice versa was also used to test the interdependency of these subunits. Lamin A/C is included as a negative control, GAPDH as a loading control. Molecular-mass markers are indicated (kDa). (H,I) Results were analyzed to determine whether depletion of both WDR34 and WDR60 simultaneously could enhance the phenotypes seen in terms of (H) the percentage of cells producing cilia or (I) the length of remaining cilia. In both cases no additive effect was seen. Data in D,E,H,I show the mean±s.d.; *P<0.05; **P<0.01; ***P<0.001. (J) Distribution of cilium length represented as a cumulative frequency chart of the percentage of total cilia found in 0.25-µm bins. The data plotted are the same as that shown in I. Scale bars: 10 µm.

We have shown previously that defects in cilia formation and function are evident following depletion of Tctex-1 (Palmer et al., 2011) and WDR34 (Asante et al., 2013) *in vitro*. Specifically, siRNA-mediated depletion of either of these proteins results in a reduced number of cells that produce cilia but also a lengthening of those cilia that remain. To determine whether this was true of the newly identified dynein-2 subunits, we first examined the role of WDR60. The biochemical data shown above suggest that WDR34 and WDR60 might act within the same dynein-2 complex. We found that endogenous WDR60, like WDR34, also localized around the centrosome (Fig. 4A) and that this localization was lost following depletion of either WDR60 itself or of WDR34 (Fig. 4A), suggesting that they function together. Consistent with this, depletion of either WDR34 or WDR60 resulted in a loss of WDR34 labeling around the centrosome (Fig. 4B). Depletion of WDR60 clearly led to one of two phenotypes – an increase in cilia length (example shown in Fig. 4C for WDR60 siRNA #1) or a complete loss of an extended axoneme (example shown in Fig. 4C for WDR60 siRNA #2), both of which were associated with a loss of labeling of WDR60 (Fig. 4C). Quantification of these data showed that loss of WDR60 resulted in a significant reduction in the proportion of ciliated cells (Fig. 4D), with a clear increase in length of the remaining cilia (Fig. 4E); this is highly similar to what we observed following depletion of WDR34 (Asante et al., 2013). The increase in cilia length is particularly evident on a cumulative frequency plot (Fig. 4F). Immunoblotting confirmed the efficacy of WDR34 and WDR60 siRNAs (Fig. 4G), as well as revealing that WDR34 and WDR60 were each reciprocally required for stability of the other. We then repeated our analysis using both WDR34 and WDR60 siRNAs. Of note, double depletion of both WDR34 and WDR60 did not further exacerbate the cilia phenotypes seen (Fig. 4H–J).

### The localization of WDR60, like that of WDR34, is dependent on giantin

**Fig. 5.**
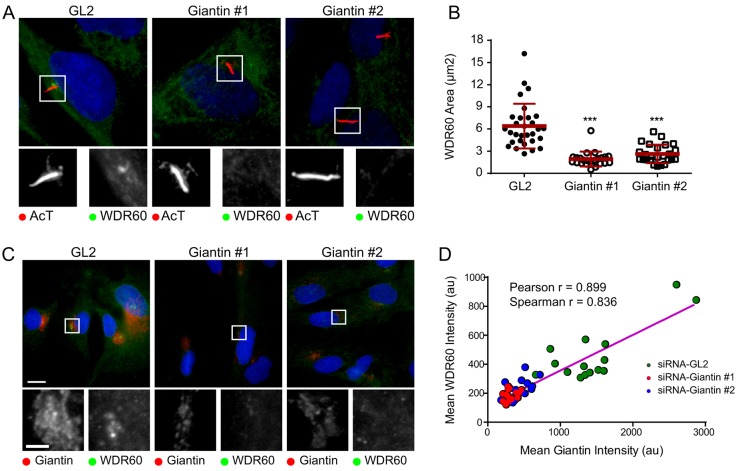
**Suppression of giantin results in a loss of pericentrosomal WDR60.** (A) Cells depleted of giantin were labeled to detect acetylated tubulin (AcT) and WDR60. (B) The area of pericentrosomal WDR60 labeling was measured and plotted. Data show the mean±s.d.; ***P<0.001. au, arbitrary units. (C) The loss of pericentrosomal WDR60 labeling correlates with the efficacy of giantin suppression. In A,C, the areas outlined are shown at higher magnification below the main image. (D) Data were quantified and the correlation was tested using both Pearson's and Spearman coefficients. Color coding indicates siRNA transfection as indicated. Scale bars: 10 µm.

In our previous work, we showed that the localization of WDR34 to the pericentrosomal region was dependent on the function of the transmembrane Golgi matrix protein giantin (also known as GOLGB1) (Asante et al., 2013). If WDR34 and WDR60 are indeed in complex together then one would predict the same would hold true for WDR60. Fig. 5A shows that loss of giantin by siRNA-mediated depletion does indeed result in a reduction in the amount of WDR60 labeling at the centrosome (quantified in Fig. 5B). The degree of loss of giantin also correlated with the intensity of labeling of WDR60 in the pericentrosomal area (Fig. 5C, quantified in 5D) as we have seen previously for WDR34. The siRNA duplexes used to target giantin have been characterized previously (Asante et al., 2013), with siRNA #1 being more effective than siRNA #2. This also is evident from the data seen in Fig. 5D.

### NudCD3 is required for the localization of WDR34

**Fig. 6.**
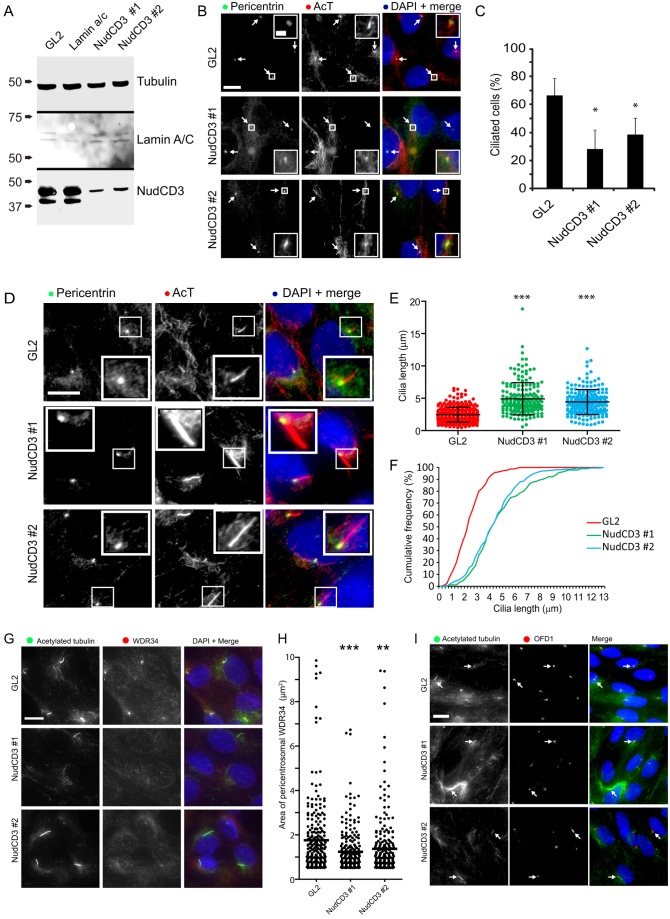
**NudCD3 is required for ciliogenesis and cilia length control.** Two independent siRNA duplexes targeting NudCD3 were used. (A) Immunoblots show tubulin as a loading control, lamin A/C as an siRNA control and NudCD3. (B) Cells were immunolabeled to detect pericentrin and acetylated tubulin (AcT). Arrows indicate examples of cilia emerging from centrosomes. Enlargements highlight the failure to extend cilia from the basal body in NudCD3-depleted cells. (C) The proportion of ciliated cells was quantified. (D) Further examples of NudCD3-depleted cells show those in which cilia are evident. (E) Quantification shows that in cells depleted of NudCD3 that extended cilia, these were longer than those seen in control cells. Data in C,E show the mean±s.d. (F) Distribution of cilium length represented as a cumulative frequency chart of the percentage of total cilia found in 0.25-µm bins. The data plotted are the same as that shown in E. (G) Depletion of NudCD3 results in a loss of pericentrosomal WDR34 (red) as shown by immunofluorescence. The centrosome and cilia are labeled with acetylated tubulin. (H) Data were quantified and the mean is shown by the horizontal line; *P<0.05; **P<0.05; ***P<0.01. (I) Suppression of NudCD3 does not affect the accumulation of OFD1 (red) around the centrosome (acetylated tubulin, green). Arrows indicate examples of cilia emerging from centrosomes. Scale bars: 10 µm.

We then used similar assays to test the role of NudCD3 in ciliogenesis. We suppressed the expression of NudCD3 using siRNA (Fig. 6A) and found that this diminished the ability of cells to generate primary cilia (Fig. 6B, quantified in 6C). Furthermore, those cilia that were seen to remain were found to be longer (Fig. 6D, quantified in 6E with cumulative frequency plots shown in 6F). Consistent with our other data, depletion of NudCD3 also resulted in a loss of WDR34 around the pericentrosomal region (Fig. 6G; quantified in Fig. 6H) without affecting the localization of OFD1 (Fig. 6I) or pericentrin (Fig. 6B).

### TCTEX1D2 is required for normal cilia function

**Fig. 7.**
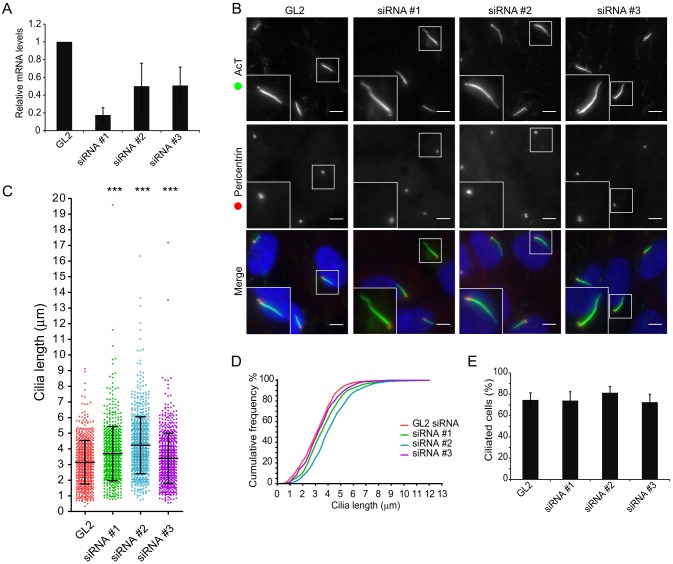
**TCTEX1D2 depletion increases cilia length.** RPE1 cells were depleted of TCTEX1D2 using three different siRNA duplexes as indicated and then serum starved for 24 h prior to (A) analysis by QPCR or (B–D) fixation and immunolabeling to detect acetylated tubulin (AcT) and pericentrin. (A) Relative TCTEX1D2 mRNA expression in transfected cells, as determined by RT-PCR. Data show the mean±s.e.m.; *n* = 3. (B) Representative maximum intensity *z*-stack projections of control and depleted cells. Enlargements highlight the increase in cilia length upon TCTEX1D2 depletion. Scale bars: 10 µm. (C) Dot plot comparing cilia lengths in control and TCTEX1D2-depleted cells. Data show the mean±s.d.; *n* = 3; ***P<0.001. (D) Distribution of cilium length represented as a cumulative frequency chart of the percentage of total cilia found in 0.25-µm bins. The data plotted are the same as that shown in C. (E) The percentage of ciliated cells found in control and TCTEX1D2-depleted cells. There is no consistent change following siRNA treatment. Data show the mean±s.e.m.

We then examined the role of TCTEX1D2 using siRNA transfection. Fig. 7A shows the efficacy of three individual siRNAs, as measured by quantitative PCR (QPCR). As shown in Fig. 7B and quantified in Fig. 7C, depletion of TCTEX1D2 results in longer cilia following serum withdrawal. In all cases, we can detect a statistically significant increase in cilia length following TCTEX1D2 depletion. This is more evident in cumulative frequency plots (Fig. 7D). Notably, the ability of cells to form primary cilia is not obviously affected following TCTEX1D2 depletion (Fig. 7E).

## DISCUSSION

**Fig. 8.**
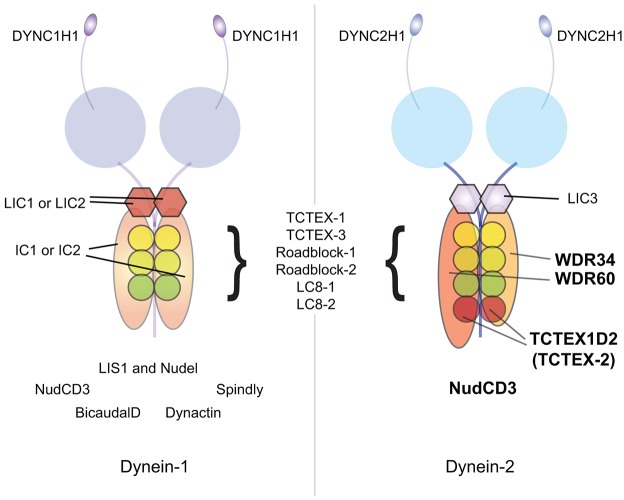
**Schematic of cytoplasmic dynein-1 and dynein-2 complexes.** Light intermediate chains are shown as hexagons, intermediate chains as extended ovals and light chains as circles. Additional interacting partners are seen for dynein-1 (left) that are not associated with dynein-2 (right). NudCD3 associates with both cytoplasmic dynein complexes. It remains unclear whether TCTEX1D2 is present as a monomer or dimer within the complex.

Here, we provide the first molecular characterization of the human dynein-2 complex. Our data build on previous reports to provide a comprehensive picture of the subunit composition of this motor, as summarized in the schematic in Fig. 8. Our data show that human dynein-2 contains both WDR34 and WDR60 intermediate chain subunits. Individual cytoplasmic dynein-1 complexes contain two copies of the same intermediate chain and same light intermediate chain (typically DYNC1I2 in most cells, DYNC1I1 in neurons). Biochemical experiments using *Chlamydomonas* have shown that FAP133 (WDR34) co-purifies and co-immunoprecipitates with other dynein-2-specific (heavy chain and light intermediate chain) subunits and with the dynein light chain, LC8. These data are consistent with FAP133 being a dynein-2 intermediate chain subunit (Rompolas et al., 2007), and they led the authors to propose a model in which dynein-2 contains two copies of FAP133, analogous to the dynein-1 complex containing two copies of the intermediate chain. Our data suggest that, in fact, dynein-2 contains both WDR34 and WDR60 intermediate chains. This asymmetry has the potential to provide more functional specialization and greater control of motor function. Furthermore, WDR60 is a larger protein than either WDR34 or the IC subunits of dynein-1. It is tempting to speculate that this asymmetry is related to its function. Evidence, primarily from *Chlamydomonas*, demonstrates that axonemal dyneins contain multiple intermediate chains within the same complex (Mitchell and Rosenbaum, 1986; King et al., 1991). For example, inner arm dynein I1 contains three intermediate chain subunits IC140, IC138 and IC97. Dimeric outer arm dynein from *Chlamydomomas* contains two intermediate chain subunits IC1 and IC2. As such, cytoplasmic dynein-2 shows similarities to axonemal dyneins. This could relate to the association of these motors with axonemal microtubules.

It is interesting to note that although mGFP–WDR34 is detectable in cilia, labeling with currently available antibodies does not detect this pool (see also Asante et al., 2013; Schmidts et al., 2013a). These data suggest that the antibody epitope is occluded within cilia. This could be due to dynein binding to the axonemal microtubules or to other factors, such as the IFT-A particle. The pericentrosomal labeling could indicate a different set of associated factors at the cilia base or that dynein-2 is assembled at the base of primary cilia, exposing a pool of WDR34. We also cannot rule out that WDR34 itself has some function outside of the context of the assembled dynein-2 complex; our gel filtration data are consistent with the presence of mGFP–WDR34 within or in association with high-molecular-mass complexes. On the basis of our co-immunoprecipitation data, we argue in favor of all WDR60 being in association with WDR34. Sucrose density gradient centrifugation shows that there is a proportion of WDR34 outside of the dynein-2 complex that could act in isolation, in a heterodimeric complex with WDR60 or in some other complex. Our gel filtration experiments are also consistent with this. Although we have not been able to monitor the dynein-2 heavy chain by immunoblotting, our previous work with dynein-1 has shown that loss of the intermediate chain subunit did not result in concomitant loss of the heavy chain (Palmer et al., 2011). These data also therefore do not rule out the possibility of a functional dynein-2 heavy chain without associated WDR34 and WDR60. However, the importance of these intermediate chain subunits for dynein-2 function is underscored by their conservation through evolution (Wickstead and Gull, 2007). Mutation of either WDR34 (Huber et al., 2013; Schmidts et al., 2013a) or WDR60 (Rompolas et al., 2007) causes skeletal ciliopathies with a similar phenotype to those seen following mutation of the dynein-2 heavy chain (Dagoneau et al., 2009; Schmidts et al., 2013b). The findings that WDR34 cannot compensate for loss of function of WDR60 and vice versa are consistent with our model that both act together in the same dynein-2 complex. We conclude that the full dynein-2 complex, including both WDR34 and WDR60, operates in IFT within cilia.

Analysis of dynein light chain function in the context of cilia biology provides important clues to the way in which dynein-2 assembles and controls protein distribution within (and retrograde exit from) cilia. The association of Tctex-1 with dynein-2 complexes was predicted from our previous work (Palmer et al., 2011). Here, we now also identify the TCTEX1D2 light chain (Tctex-2) as a light chain for dynein-2, not present in dynein-1. Tctex-2 has previously been found to control outer arm dynein assembly in *Chlamydomonas* flagella (Patel-King et al., 1997; Pazour et al., 1999). Evidence suggests that, unlike other dynein light chains, TCTEX1D2 is monomeric (DiBella et al., 2001). One possibility is that a single copy of TCTEX1D2 binds to WDR34, with an alternative light chain (or a functionally similar protein) binding to WDR60. This could diversify the regulation of dynein-2 function and potentially influence the cargo-binding capabilities of the complex. Our data show that a reduction in TCTEX1D2 expression results in an increase in cilia length similar to that which we observed following depletion of WDR34, WDR60 and giantin. Of note, TCTEX1D2-depleted cells still form cilia. We cannot reject the idea that TCTEX1D2 is required for cilia formation, as these experiments are only depletion experiments and not a complete knockout. The moderate phenotype seen here *in vitro* might also reflect some functional overlap with other dynein light chains in this system. It remains unclear whether dynein light chains act in directing dynein complex assembly and/or in cargo binding. It is also important to note that our *in vitro* assays reporting an elongation of cilia do not reflect the situation *in vivo* where the primary failure appears to be in ciliogenesis, i.e. the formation of the axoneme. Our interpretation is that our siRNA experiments reflect a defect in retrograde IFT.

NudCD3 has been shown to influence the assembly and stability of the dynein-1 complex (Zhou et al., 2006), and it localizes to centrosomes (Cai et al., 2009). Our data are consistent with NudCD3 playing a similar role with dynein-2. We have not been able to detect NudCD3 in cilia by immunofluorescence or using epitope-tagged forms (Zhou et al., 2006), which suggests that this protein plays a role in the assembly of dynein-2 within the main body of the cell. The association of NudCD3 with both cytoplasmic dynein complexes makes the analysis of its function more complex. Its role in ciliogenesis is most likely linked to its interaction with dynein-2. However, we would not expect to find mutations in this gene associated with ciliopathies, as a loss-of-function would compromise dynein-1, likely leading to cell death. We also identified the related NudC protein in our proteomics analysis (supplementary material Table S1C) but, as of yet, we have not investigated further the role of NudC in cilia or dynein-2 function; however, we note that NudC has been shown to localize to motile cilia (Pedersen et al., 2007).

In our experiments we typically observed two outcomes following siRNA-mediated suppression of dynein-2 subunits – there were fewer cilia but those that remained were longer. We interpret this as a threshold effect of our *in vitro* experiments. Partial loss of function compromises retrograde IFT and results in longer cilia (as anterograde IFT continues to deliver axonemal and other components to the tip). More effective depletion results in an inability of cells to form cilia, with limited or no extension of the axoneme. It is also important to note that longer cilia have not typically been described for patient mutations; indeed, cells from patients with WDR34 mutations show shorter cilia on average (Huber et al., 2013). However, phenotypic variability, with some cells failing to extend an axoneme while others can, has been seen in fibroblasts from patients with WDR60 mutations (McInerney-Leo et al., 2013). This distinction between *in vitro* and *in vivo* outcomes necessitates caution in the interpretation of the data. Nonetheless, our data show clear roles for WDR34, WDR60 and TCTEX1D2 within the context of the dynein-2 complex.

It is also significant to note that we do not detect any association of dynein-2 with many known regulators of cytoplasmic dynein-1 and axonemal dynein, including dynactin and LIS1. It is, however, important to note that although our work does show that dynactin and LIS1 do not stably associate with dynein-2, our data do not preclude that they do not associate transiently with dynein-2. For example, LIS1 acts in the initiation of dynein-1 motility but does not remain associated with it during organelle transport (Egan et al., 2012). Consistent with such a model, although we do not detect LIS1 in association with dynein-2 by co-immunoprecipitation, we can localize LIS1 to primary cilia by immunofluorescence (data not shown). LIS1 is also believed to act in generating a persistent force state of dynein to aid its function in moving heavy loads (McInerney-Leo et al., 2013). Although such functions are required of dynein-1 (for example in nuclear migration) and axonemal dyneins (in generating the force of cilia and flagellar beating), it is conceivable that such large cargo loads are not part of the cargo repertoire transported by dynein-2. LIS1, dynactin and BICD family proteins likely cooperate to direct the association of dynein with cargo and subsequent motility (Egan et al., 2012; Splinter et al., 2012; Wang et al., 2013; Moon et al., 2014). The absence of dynactin suggests that other as-yet-unidentified accessory factors likely act in cooperation with dynein-2 in linking the motor to its cargo. Other recent *in vitro* reconstitution experiments have shown that dynein-1 requires the association of both dynactin and another accessory factor such as BICD2 to induce a processive state (McKenney et al., 2014; Schlager et al., 2014). Dynein-2 is also likely to be a processive motor, and it is tempting to speculate that additional factors are required to induce processivity, possibly the IFT-A complex itself. Further biochemical and biophysical analysis would undoubtedly help to address such questions. In conclusion, our characterization of the subunit composition of cytoplasmic dynein-2 defines a requirement for both WDR34 and WDR60 in dynein-2 function, explaining why mutations in either gene cause disease. Furthermore, this work identifies TCTEX1D2 as a candidate ciliopathy gene.

## MATERIALS AND METHODS

All reagents were purchased from Sigma-Aldrich (Poole, UK) unless otherwise stated.

### Growth of culture cells

Human telomerase-immortalized retinal pigment epithelial cells (hTERT-RPE1) were maintained in DMEM-F12 supplemented with 10% FCS (Life Technologies, Paisley, UK) supplemented with 1% L-glutamine and 1% essential amino-acids. Cells were seeded onto 35-mm glass-bottomed dishes (MatTek, Ashland, MA).

### Ciliogenesis assay

On reaching confluence, hTERT-RPE1 cells were rid of serum by washing twice with PBS and were incubated at 37°C under 5% CO_2_ for 24–48 h in serum-free medium to induce cell cycle exit and subsequent cilium assembly. Cells were fixed and typically labeled with anti-acetylated tubulin to mark primary cilia. Cilia lengths were measured using the Fiji implementation of Image J (Schindelin et al., 2012).

### Antibodies

Antibodies used in this study include: rabbit polyclonal anti-WDR60 (HPA020607), rabbit polyclonal anti-NudCD3 (HPA019136), rabbit polyclonal anti-ODF2 (HPA001874), mouse monoclonal anti-acetylated tubulin (T6793) and mouse monoclonal anti-γ-tubulin (T6557) (all from Sigma-Aldrich); rabbit polyclonal anti-giantin (PRB-114C), mouse monoclonal anti-GFP (MMS-118P) and rabbit polyclonal anti-pericentrin (PRB-432C) (all from Covance, CA); mouse monoclonal anti-p150-glued (610473) and mouse monoclonal anti-p50-dynamitin (BD 611003) (both from BD Biosciences, Oxford, UK); rabbit polyclonal anti-WDR34 (NBP1-88805, Novus Biologicals, Cambridge, UK); rabbit polyclonal anti-Tctex1 (sc-28537, Santa Cruz Biotechnology, Dallas, TX); mouse monoclonal anti-GAPDH (ab9484) and rabbit polyclonal anti-TCTEX1D2 (ab139804) (both from Abcam, Cambridge, UK); mouse monoclonal anti-DIC74 (MAB1618, Millipore, Feltham, UK); rabbit polyclonal anti-Lamin A/C (2032, Cell Signaling Technologies, Hitchin, UK); rabbit polyclonal anti-OFD1 (a generous gift from Andrew Fry, University of Leicester, UK); and rabbit polyclonal anti-LIS1 (A300-409A, Bethyl Laboratories, from Cambridge Bioscience, Cambridge, UK). Cy2-conjugated donkey anti-mouse-IgG (715-225-151), Cy3-conjugated donkey anti-rabbit-IgG (711-165-152), peroxidase-conjugated donkey anti-mouse-IgG (715-035-150) and peroxidase-conjugated donkey anti-rabbit-IgG (711-035-150) were supplied by Jackson ImmunoResearch (from Stratech Scientific, Newmarket, UK). IRDye 800CW donkey anti-mouse-IgG (926-32212) and IRDye 680 donkey anti-rabbit-IgG (926-32223) were from Li-Cor (Cambridge, UK).

### Cloning of mGFP-WDR34, generation of lentiviruses and stable cell lines

The human WDR34 gene (RefSeq: NM_052844.3) was amplified from the Origene cDNA (SC319901, Cambridge Bioscience) by PCR using the Phusion^TM^ high-fidelity DNA polymerase PCR kit (New England BioLabs, Hitchin, UK) with the forward primer 5′-GGAACTCGAGATGGCAACCCGCGCGCAGCC-3′ and the reverse primer 5′-GAAAGAATTCTCAGGCCGCCACCTCTGCTGC-3′, containing the *Xho*I and *Eco*RI restriction sites, respectively. The PCR product was subcloned into a modified pLVX-Puro vector (Clontech, Mountain View, CA) that includes the mGFP sequence flanked by *Xho*I and *Eco*RI restriction sites. The resultant pLVX-Puro-mGFP-WDR34 cDNA was amplified in XL10-Gold ultracompetent bacterial cells (Stratagene) and harvested from the bacterial cells using QIAprep Spin Miniprep Kit (Qiagen, Manchester, UK). All plasmid minipreps were validated by restriction analysis and subsequently sequenced to facilitate secondary validation by sequence alignment analysis.

Lentiviral particles of pLVX-Puro-mGFP-WDR34 were produced in HEK293T cells using the Lenti-X^TM^ HTX Packaging System (Clontech), and low passage hTERT-RPE1 cells were transduced with the resultant viral supernatant, strictly according to the manufacturer's directives. At 48 h post-transduction, confluent cells were subcultured into 10%-FBS-supplemented DMEM F-12 HAM (Sigma-Aldrich, Poole, UK) containing 5 µg/ml puromycin. Cells were maintained in 0.25 µg/ml puromycin after this.

### Proteomics

Three 15-cm dishes each of hTERT-RPE1-mGFP (control) and hTERT-RPE1-mGFP-WDR34 cells were serum-starved for 24 h at confluence to induce ciliogenesis. Cells were briefly washed twice with 20 ml of ice-cold PBS per dish, and the PBS was drained off completely by leaning the dishes at ∼60° for ∼10 s. 500 µl of ice-cold lysis buffer 1 [10 mM Tris-HCl pH 7.4, 50 mM NaCl, 0.5 mM EDTA, 1.0% IGEPAL (CA-630, Sigma)], containing freshly added 1 mM phenylmethane sulfonylfluoride (PMSF) and 1× EDTA-free Protease Inhibitor Cocktail Set V (539137, Millipore, UK), was added to the cells, and the cells were scraped off the dish into 2-ml tubes. Cells were lysed by incubation with the buffer on a rotor at 4°C for 30 min. Lysates were centrifuged at 20,000 ***g*** at 4°C for 10 min, and supernatants were transferred into pre-cooled 2-ml Eppendorf tubes.

### Equilibration of GFP nano-trap beads and immunoprecipitation

A total of 20 µl of resuspended GFP nano-trap beads (Chromotek, Planegg-Martinsried, Germany) per sample was added to 500 µl of ice-cold dilution buffer (10 mM Tris-HCl pH 7.4, 50 mM NaCl, 0.5 mM EDTA, freshly added 1 mM PMSF and 1× EDTA-free Protease Inhibitor Cocktail), spun down at 2700 ***g*** for 2 min at 4°C, and the supernatant discarded. This wash was repeated two more times. The cell lysate supernatants were added to the equilibrated beads and incubated on a rotor at 4°C for 2 h.

### Sample preparation for mass spectrometry

At the end of the incubation, the tubes were centrifuged at 2000 ***g*** for 2 min at 4°C, and the supernatants discarded. A total of 500 µl of ice-cold dilution buffer was added to the protein-bound beads (pellets), the tubes were inverted gently approximately ten times to resuspend the contents and spun down at 2000 g for 2 min at 4°C. This wash was repeated two more times, after which the beads were resuspended in 50 µl of 2× lithium dodecyl sulphate (LDS) sample buffer (NP0007, Life Technologies, Paisley, UK) (106 mM Tris-HCl, 141 mM Tris-base, 2% LDS, 10% glycerol, 0.51 mM EDTA, 0.22 mM SERVA® Blue G-250 and 0.175 mM Phenol Red pH 8.5), containing 1× Nupage® sample reducing agent (500 mM dithiothreitol) (NP0004). The beads were boiled at 95°C for 10 min to denature proteins and dissociate precipitated proteins from the GFP nano-trap beads, and the tubes were immediately placed on ice for 1 min to condense the vaporized contents. The tubes were centrifuged at 2700 ***g*** for 2 min at 4°C to sediment the beads, and the supernatants were collected into fresh 1.5-ml Eppendorf tubes. These samples were submitted to the proteomics facility at the University of Bristol for mass spectrometry analysis.

### Mass spectrometry

Samples were run on a 10% acrylamide gel to separate proteins. Each gel lane was cut into six slices and each slice was subjected to in-gel tryptic digestion in a ProGest automated digestion unit (Digilab, UK). An Ultimate 3000 nano HPLC system operated in line with an LTQ-Orbitrap Velos mass spectrometer (Thermo Scientific) was employed in fractionating the resulting peptides. Summarily, peptides in 1% (v/v) formic acid were injected onto an Acclaim PepMap C18 nano-trap column (Thermo Scientific). The peptides were washed with 0.5% (v/v) acetonitrile in 0.1% (v/v) formic acid and resolved on a 250-mm×75-µm Acclaim PepMap C18 reverse phase analytical column (Thermo Scientific) over a 150-min organic gradient composed of seven gradient segments. Peptides were run over the column through the following sequential gradients: 1–6% solvent B for 1 min (solvent B – aqueous 80% acetonitrile in 0.1% formic acid; solvent A – 0.1% formic acid), 6–15% solvent B for 58 min, 15–32% solvent B for 58 min, 32–40% solvent B for 5 min, 40–90% solvent B for 1 min, 90% solvent B for 6 min and, finally, 1% solvent B for 1 min; with a flow rate of 300 nl/min. Peptides were subjected to 2.1-kV nano-eloctrospray ionisation from a 30-µm (internal diameter) stainless steel emitter (Thermo Scientific) with a capillary temperature of 250°C. An LTQ-Orbitrap Velos mass spectrometer operated by the Xcalibur 2.1 software (Thermo Scientific) in data-dependent acquisition mode was used to acquire tandem mass spectra of the peptides. Survey scans were analyzed at 60,000 resolution (at m/z 400) within the mass range m/z 300–2000, and the top 20 multiple-charged ions in each duty cycle were selected for MS/MS in the LTQ linear ion trap. Charge-state filtering, which excludes unassigned precursor ions from fragmentation, and dynamic exclusion (repeat count, 1; repeat duration, 30 s; exclusion list size, 500) were applied. The following fragmentation conditions were set in the LTQ: 40% normalized energy of collision, 0.25 activation q, 10-ms activation time and a minimum ion selection intensity of 500 counts.

Proteome Discoverer software (version 1.2, Thermo Scientific) was used to process and quantify the raw data files, which were then searched against the UniProt human database (122,604 sequences) using the SEQUEST algorithm (version 28, revision 13). Tolerances of 10 ppm and 0.8 Da were set for peptide precursor mass and MS/MS, respectively. Cysteine carbamidomethylation (+57.0214) and methionine oxidation were included in the search criteria as fixed modifications and variable modifications, respectively. Searches were performed with full tryptic digestion allowing a maximum of 1 missed cleavage. All peptide data was filtered to satisfy a 5% false discovery rate (FDR) by enabling the reverse database search option. The Proteome Discoverer software creates a reverse ‘decoy’ database from the same protein database; peptides evading the initial filtering parameters that were derived from this decoy database are badged as false-positive identifications. The minimum cross-correlation factor (Xcorr) filter was separately readjusted for each individual charge state to meet the predetermined target 5% FDR, considering the number of random false-positive matches from the reverse decoy database. Those proteins identified in supplementary material Table S1 were selected on the basis of being known components or well-characterized interactors of cytoplasmic dynein-1.

### GFP-trap immunoprecipitation

Cells were grown to confluence in 15-cm dishes and serum starved for 24 h. Cells were washed twice with ice-cold PBS, lysed with 500 µl of ice-cold buffer (10 mM Tris-HCl pH 7.4, 50 mM NaCl, 0.5 mM EDTA, 1.0% IGEPAL CA-630, 1 mM PMSF and 1× protease inhibitor cocktail) on a rotator for 30 min at 4°C, and the lysate supernatant was collected after centrifuging at 20,000 ***g*** for 10 min at 4°C. Lysate supernatants were incubated with equilibrated GFP nano-trap beads (Chromotek) on a rotator for 90 min at 4°C, after which the beads were washed three times with 500 µl of dilution buffer (10 mM Tris-HCl pH 7.4, 50 mM NaCl, 0.5 mM EDTA, 1 mM PMSF and 1× protease inhibitor cocktail) by centrifuging at 2000 ***g*** for 2 min at 4°C. Beads were resuspended in 50 µl of 2× LDS sample buffer (Life Technologies) containing sample reducing agent (Life Technologies) and boiled at 95°C for 10 mins, followed by SDS-PAGE and immunoblotting as described below.

### Small interfering RNA transfection

Cells were siRNA-transfected by using the calcium phosphate method at 3% CO_2_ (Chen and Okayama, 1988). The medium was changed at 20 h post-transfection, and cells were washed with PBS and were incubated for 72 h (at 37°C under 5% CO_2_) with fresh supplemented medium. siRNA duplexes were designed using online algorithms (MWG-Eurofins, Ebersberg, Germany) and were subsequently synthesized by MWG-Eurofins. A BLAST search was performed for these duplexes against the non-redundant database to determine their specificity. Lamin A/C or luciferase GL2 were depleted as targeted controls. The sequences used were as follows: giantin was depleted with giantin siRNA #1 (5′-ACUUCAUGCGAAGGCCAAATT-3′) and giantin siRNA #2 (5′-AGAGAGGCUUAUGAAUCAATT-3′). Duplexes for suppressing WDR34 were WDR34 #1 (5′-GAUGGUGUCUUGUCUGUAU-3′) and WDR34 #2 (5′-GCUGUUUGAUCUCCAGAAA-3′). WDR60 was targeted with WDR60 #1 (5′-CCAUUUGGAGAACCAAUAU-3′) and WDR60 #2 (5′-CAUGGUAUAAGACCAGUGA-3′). Duplexes targeting NUDCD3 included NUDCD3 #1 (5′-GUGAUGCAGUGGUGUGAGA-3′) and NUDCD3 #2 (5′-GAGAAGGCAGGAACUUGAA-3′). Luciferase GL2 (5′-CGUACGCGGAAUACUUCGAUU-3′) and lamin A/C (5′-CUGGACUUCCAGAAGAACA-3′) were used as targeted negative and positive controls, respectively. TCTEX1D2 was depleted with TCTEX1D2 #1 (5′-AGAGGUGAAGGAGUAUUCATT-3′), TCTEX1D2 #2 (5′-UGCUGAAUAUUCUCCAGAATT-3′) and TCTEX1D2 #3 (targeting the 3′UTR; 5′-AGGACAUGACCAUGAAGAATT-3′).

### Quantitative PCR

RNA was extracted from transfected cells using a Qiagen RNeasy purification kit according to the manufacturer's instructions (Qiagen, Manchester, UK). cDNA was generated using an SuperScript^TM^ III First Strand Synthesis System (Life Technologies). DNA was amplified under the following conditions: 95°C for 10 min then 30 cycles of 95°C for 30 s, 55°C for 30 s, 70°C for 15 s. Amplification was monitored by incorporation of SYBR® Green (Finnzyme, Espoo, Finland) and analyzed on a BioRad Opticon 2 PCR system (BioRad, Hemel Hempstead, UK). Gene expression was quantified by the ΔΔCT method (Livak and Schmittgen, 2001) normalizing against GAPDH. Single-product amplification was verified by performing melting curve analysis and gel electrophoresis. The primers (MWG Eurofins) used were as follows: TCTEX1D2 forward primer, 5′-GGAGCCCGAGAACACCTATATT-3′; reverse primer, 5′-GCTGAGGCATTTCTTCTGGAGA-3′; GAPDH forward primer, 5′-ATCCCATCACCATCTTCCAG-3′; reverse primer, 5′-CCATCACGCCACAGTTTCC-3′.

### Sucrose density gradient centrifugation

Cells were grown and lysed as described for the GFP-trap experiment and lysate supernatants were layered onto 5–40% or 5–20% continuous sucrose gradient columns. The former were poured using a Perspex gradient mixer, the latter using Biocomp isopycnic gradient forming tube caps (10-mm isopycnic long caps, Biocomp; from Wolf Laboratories, Manchester, UK). Gradients were centrifuged at 96,119 ***g*** for 18 h at 4°C in a TH-641 swinging bucket rotor (Thermo Scientific). Proteins were precipitated with 250 µl or 125 µl of 100% trichloroacetic acid at 4°C for 1 h from 1-ml or 500-µl column fractions, respectively, pelleted by centrifuging at 16,168 ***g*** for 5 min at 4°C, and washed three times with 150 µl of ice-cold acetone. The acetone was chased off by incubation at room temperature for 30 min, and pellets were resuspended in 1× LDS sample buffer containing reducing agent.

### Immunoblotting

For immunoblots to validate siRNA efficacy, cells were lysed and samples were separated by SDS-PAGE followed by transfer to nitrocellulose membranes; primary antibodies were detected by using HRP-conjugated secondary antibodies (Jackson ImmunoResearch, West Grove, PA) and enhanced chemiluminescence (ECL, GE Healthcare, Cardiff, United Kingdom). Immunoblots in Fig. 6 were developed using an Odyssey Sa imager (Li-Cor, Cambridge, UK).

### Gel filtration

The following buffer was used for gel filtration: 30 mM HEPES (pH 7.0), 150 mM KOAc, 2 mM MgSO_4_, 0.68 M glycerol and 2 mM DTT. A 24-ml Superose 6 column (GE Healthcare) was calibrated using 70S ribosome, apoferritin, β-amylase, BSA and carbonic anhydrase. Cells expressing either GFP or mGFP–WDR34 were lysed at 4°C using a ball bearing cell homogenizer with 10-µm clearance (Isobiotec, Heidelberg) in gel filtration buffer, and the lysates were cleared by centrifugation for 30 min at 25,000 ***g***. Fractions (0.5 ml each) were collected and the fluorescence of each sample was measured using a Cary Eclipse Fluorescence Spectrophotometer (Agilent Technologies, Santa Clara, CA) at 25°C with excitation at 490 nm (5 nm excitation slit) and emission scanning 500–600 nm with 5-nm emission slits.

### Immunolabeling and microscopy

Medium was removed and cells were subsequently washed with PBS. Cells were then fixed using cold methanol for 4 min at −20°C. For images in Fig. 1E,F, cells were fixed with 4% paraformaldehyde in PBS, permeabilized with 0.1% Triton X-100 for 5 min and then washed in PBS. After two washes in PBS, cells were blocked using 3% BSA in PBS for 30 min at room temperature. Three washes with PBS of 5 min each at room temperature were performed after each of the primary and secondary antibody incubations. Nuclear staining was performed using DAPI [4,6-diamidino-2-phenylindole (Life Technologies), diluted at 1∶5000 in distilled water] for 3 min at room temperature, and the cells were then rinsed twice in PBS. Cells were imaged using an Olympus IX-71 or IX-81 widefield microscope with a 63× 1.42 N.A. objective, and excitation and emission filter sets (Semrock, Rochester, NY) controlled by Volocity software (version 4.3, Perkin-Elmer, Seer Green, UK).

### Processing and quantification of image data and statistical analysis

Representative images are shown, and all experiments were repeated independently at least three times each. Samples were compared using the Kruskal–Wallis one-way analysis of variance with Dunn's post-hoc test using GraphPad Prism version 4. All images were prepared with Adobe Photoshop and Adobe Illustrator. All data and resulting statistical analyses arise from three independent experiments.

## Supplementary Material

Supplementary Material
