# Supplementary material for: Subunit composition of the human cytoplasmic dynein-2 complex
Source: J Cell Sci. 2014 Nov 1;127(21):4774–87. doi: 10.1242/jcs.159038 (PMC4215718; doi:10.1242/jcs.159038)
Supplement: Supplementary Material [file supp_127_21_4774__index.html]

Subunit composition of the human cytoplasmic dynein-2 complex — Supplementary Material 

# Subunit composition of the human cytoplasmic dynein-2 complex

## JCS159038 Supplementary Material

**Files in this Data Supplement:**

- **Supplementary Material**
