# Supplementary material for: Subunit composition of the human cytoplasmic dynein-2 complex
Source: J Cell Sci. 2014 Nov 1;127(21):4774–87. doi: 10.1242/jcs.159038 (PMC4215718; doi:10.1242/jcs.159038)
Supplement: Supplementary Material [file supp_127.21.4774_JCS159038.pdf]

TABLE 1: PROTEOMIC ANALYSIS OF MGFP-WDR34 VERSUS MGFP IN STABLY-TRANSFECTED, SERUM-STARVED HTERT-RPE1 CELLS.

TABLE 1A: CYTOPLASMIC DYNEIN-2 SUBUNITS, NOT FOUND ASSOCIATED WITH DYNEIN-1

Accession	# AAs	MW [kDa]	Description	Score mGFP	Coverage mGFP	# PSM mGFP	# Peptides mGFP	Score mGFP-WDR34	Coverage mGFP-WDR34	# PSM mGFP-WDR34	# Peptides mGFP-WDR34	Annotation
Q96EX3	536	57.8	WD repeat-containing protein 34 GN=WDR34	137.93	44.59	31	16	3732.64	79.85	873	34	DYNEIN WDR34
Q8WVS4	1066	122.5	WD repeat-containing protein 60 GN=WDR60	n.d.	n.d.	n.d.	n.d.	329.54	37.24	89	33	DYNEIN WDR60
B0I1S0	4307	492.3	DYNC2H1 variant protein GN=DYNC2H1	n.d.	n.d.	n.d.	n.d.	37.31	2.48	10	10	DYNEIN HEAVY CHAIN 2
E5RJK4	72	7.9	Cytoplasmic dynein-2 light intermediate chain GN=DYNC2LI1	n.d.	n.d.	n.d.	n.d.	5.38	20.83	1	1	DYNEIN LIC3
Q8WW35	142	16.1	Tctex1 domain-containing protein 2 GN=TCTEX1D2	n.d.	n.d.	n.d.	n.d.	110.43	45.07	24	5	DYNEIN TCTEX1D2

n.d. = not detected

TABLE 1B: DYNEIN-1 LIGHT CHAINS ALSO FOUND ASSOCIATED WITH DYNEIN-2

Accession	# AAs	MW [kDa]	Description	Score mGFP	Coverage mGFP	# PSM mGFP	# Peptides mGFP	Score mGFP-WDR34	Coverage mGFP-WDR34	# PSM mGFP-WDR34	# Peptides mGFP-WDR34	Annotation
P63172	113	12.4	Dynein light chain Tctex-type 1 GN=DYNLT1	n.d.	n.d.	n.d.	n.d.	84.63	60.18	19	4	DYNEIN TCTEX1
P51808	116	13.1	Dynein light chain Tctex-type 3 GN=DYNLT3	n.d.	n.d.	n.d.	n.d.	7.97	23.28	3	2	DYNEIN TCTEX3
A4D1D1	297	32.5	Similar to dynein, cytoplasmic, light peptide; 8kD LC; dynein LC8; protein inhibitor of neuronal nitric oxide synthase GN=LOC392067	n.d.	n.d.	n.d.	n.d.	4.21	7.41	2	1	DYNEIN LC8
P63167	89	10.4	Dynein light chain 1, cytoplasmic OS=Homo sapiens GN=DYNLL1	23.71	64.04	7	5	405.41	64.04	109	6	DYNEIN LIGHT CHAIN 1
Q96FJ2	89	10.3	Dynein light chain 2, cytoplasmic OS=Homo sapiens GN=DYNLL2	19.29	25.84	5	3	255.84	65.17	81	6	DYNEIN LIGHT CHAIN 2
Q9NP97	96	10.9	Dynein light chain roadblock-type 1 OS=Homo sapiens GN=DYNLRB1	52.23	55.21	16	4	709.81	85.42	202	10	DYNEIN ROADBLOCK
Q8TF09	96	10.8	Dynein light chain roadblock-type 2 OS=Homo sapiens GN=DYNLRB2	19.08	16.67	6	2	170.55	33.33	54	3	DYNEIN ROADBLOCK 2

n.d. = not detected

TABLE 1C: DYNEIN-1 ACCESSORY FACTORS ALSO FOUND IN ASSOCIATION WITH DYNEIN-2

Accession	# AAs	MW [kDa]	Description	Score mGFP	Coverage mGFP	# PSM mGFP	# Peptides mGFP	Score mGFP-WDR34	Coverage mGFP-WDR34	# PSM mGFP-WDR34	# Peptides mGFP-WDR34	Annotation
B0FTY2	361	40.8	NudC-like protein OS=Homo sapiens GN=NUDCD3	n.d.	n.d.	n.d.	n.d.	7.77	5.54	3	2	NUDCD3
Q9Y266	331	38.2	Nuclear migration protein NudC OS=Homo sapiens GN=NUDC	7.33	3.32	2	1	27.18	10.57	7	3	NUDC

n.d. = not detected

TABLE 1D: DYNEIN-1 ACCESSORY FACTORS THAT WERE NOT FOUND IN ASSOCIATION WITH DYNEIN-2

Accession	# AAs	MW [kDa]	Description	Score mGFP	Coverage mGFP	# PSM mGFP	# Peptides mGFP	Score mGFP-WDR34	Coverage mGFP-WDR34	# PSM mGFP-WDR34	# Peptides mGFP-WDR34	Annotation
G5E9H4	1139	126.7	Dynactin 1 (P150, glued homolog, Drosophila), isoform CRA_a OS=Homo sapiens GN=DCTN1	2.70	1.14	1	1	10.04	1.05	1	1	DYNACTIN
H0YIC1	228	25.4	Bicaudal D-related protein 1 (Fragment) OS=Homo sapiens GN=CCDC64	n.d.	n.d.	n.d.	n.d.	2.43	4.39	1	1	BICAUDAL D1
B4DWL0	270	30.9	Bicaudal D-related protein 1 OS=Homo sapiens GN=CCDC64	19.73	5.19	2	1	n.d.	n.d.	n.d.	n.d.	BICAUDAL D RELATED PROTEIN 1
I3L2U8	345	38.9	Platelet-activating factor acetylhydrolase IB subunit alpha OS=Homo sapiens GN=PAFAH1B1	6.44	9.28	2	2	n.d.	n.d.	n.d.	n.d.	LIS1

n.d. = not detected
